# Improved First Dose Conversion of Supraventricular Tachycardia Using Weight-Based Adenosine

**DOI:** 10.7759/cureus.35995

**Published:** 2023-03-10

**Authors:** Carina M Deck, Brian Dang, Nick Ashenburg, Brian Rice

**Affiliations:** 1 Emergency Department Pharmacy, Stanford Health Care, Palo Alto, USA; 2 Emergency Medicine, Stanford University School of Medicine, Palo Alto, USA

**Keywords:** weight-based dosing, ecg, supraventricular tachycardia, adenosine, svt

## Abstract

Introduction

Paroxysmal supraventricular tachycardia (PSVT) is an often-recurring tachyarrhythmia that frequently results in emergency department visits and is commonly treated using intravenous adenosine. Given the anecdotal variable success of adenosine, the question arose of which patient factors may affect its success. This retrospective cohort analysis seeks to test the hypothesis that adult patients who receive adenosine at doses of ≥0.1mg/kg will have greater rates of successful conversion upon receipt of the first dose of adenosine.

Methods

This retrospective cohort analysis examines the charts from patients with known paroxysmal supraventricular tachycardia from November 1, 2015, through March 31, 2020, who were treated with intravenous adenosine. The primary outcome was the first-dose success of adenosine when stratified by patient weight (greater than 0.1mg/kg or less than 0.1mg/kg). Baseline characteristics and adverse effects were also collected.

Results

Seventy-six patients were included in the analysis. Patients who received adenosine at doses greater than or equal to 0.1mg/kg were more likely to convert to sinus rhythm than those who received doses less than 0.1mg/kg (p=0.006). No difference in adverse effects was noted between the groups (p=0.75).

Conclusion

This retrospective cohort analysis found that patients who received adenosine at doses greater than or equal to 0.1mg/kg for the treatment of PSVT were more likely to convert to sinus rhythm than those who received lower doses, with no difference in adverse effects. This hypothesis-generating finding provides the basis for a subsequent randomized, controlled trial to investigate the effectiveness and safety of weight-based adenosine.

## Introduction

Supraventricular tachycardia (SVT) is a catch-all term used to describe tachycardias originating from the His bundle or above and includes atrial tachycardia, inappropriate sinus tachycardia, atrial fibrillation, junctional tachycardia, atrioventricular reentrant tachycardia (AVRT), atrioventricular nodal reentrant tachycardia (AVNRT), and other accessory pathway-mediated re-entrant tachycardias. Paroxysmal supraventricular tachycardia (PSVT) is a subtype of SVT and is characterized as a rapid, regular tachycardia of abrupt onset and termination. Diagnosis of PSVT requires a 12-lead electrocardiogram (ECG), and its rhythm is typically a regular tachycardia with a narrow QRS complex. The estimated incidence of PSVT is approximately 36 per 100,000 persons per year. For the management of regular PSVT, the 2015 AHA/ACC/HRS Guidelines for the Management of Adult Patients with Supraventricular Tachycardia recommend intravenous adenosine for initial management if vagal maneuvers are ineffective [1].

Adenosine is efficacious for PSVT by slowing the conduction time through the AV node and interrupting re-entry pathways. For adult patients, the initial recommended dose is 6mg by rapid IV bolus, followed by a rapid saline flush due to the agent’s short half-life of less than 10 seconds. If ineffective, a subsequent 12mg dose is considered. Adenosine has a high success rate, approximately 78 to 96% for AVNRT or AVRT, but first-dose success ranges widely [1-6]. For pediatrics, the recommended dosing regimen is 0.1mg/kg (maximum 6mg), and if ineffective, a second dose of 0.2mg/kg (maximum 12mg) may be considered [7]. The most reported adverse effect is chest pressure [1,8,9].

Adenosine was reported as utilized for PSVT as early as 1976 in the form of adenosine triphosphate (ATP) and was reported successful at producing conversion of PSVT at doses of 3 to 15mg in pediatric patients, and 10 to 20mg in adult patients [10,11]. Disadvantages of ATP included side effects such as flushing, malaise, headache, retching, coughing, and convulsions. These adverse effects appeared to be dose-related, and some protocols utilized lower doses of 5 to 10mg to avoid these adverse effects [11]. Adenosine later became preferred over ATP given its greater potency and improved stability [11,12]. In the pediatric literature, early reports of adenosine dosing were given in 0.05mg/kg increments, with success in terminating SVT once doses of 0.1-0.25mg/kg were given [13].

Many medications are based on weight for pediatrics, and a fixed dose for adults. Given the anecdotal variable success of adenosine in adult patients, the question arose of how patient-specific factors affect the success of adenosine for PSVT. For more than 30 years, no research had ever directly looked at correlations between body weight and adenosine. In 2022, after data collection and analysis had already been completed for our manuscript which follows, a retrospective analysis of SVT patients was published suggesting that greater weight-based doses were correlated with higher first-time sinus conversion success rates [14]. Building off of that work and the pediatric guidelines of 0.1mg/kg, we have approached fixed adenosine dosing of variable body weights by generating a natural experiment with two cohorts: those who receive greater than 0.1mg/kg, and those who receive less than 0.1mg/kg. Our objective in the following study is to use a retrospective cohort analysis to test the hypothesis that adult patients who receive a higher dose of adenosine (≥0.1mg/kg) will experience greater rates of successful conversion upon receipt of the first dose of adenosine.

## Materials and methods

Inclusion/exclusion

This study is a retrospective cohort analysis using a chart review from November 1, 2015, through March 31, 2020, from the emergency department at Stanford Health Care. At Stanford Health Care’s Adult Emergency Department, the standard of care for patients with known or suspected supraventricular tachycardia is to treat patients according to the 2015 AHA/ACC/HRS Guidelines for the Management of Adult Patients with Supraventricular Tachycardia.

Inclusion criteria were those greater than or equal to 18 years of age who had a diagnosis of confirmed PSVT and subsequent administration of intravenous adenosine. Diagnosis of PSVT was confirmed on ECG in the emergency department which was initially reviewed by an emergency medicine physician and then subsequently confirmed to be PSVT by a cardiologist. If a patient was initially treated as PSVT by the emergency physician but the rhythm on the ECG was subsequently overread by the cardiologist as anything other than PSVT, the record was excluded from the analysis. Exclusion criteria (other than those with an ECG rhythm other than PSVT) were pregnancy, prisoners, administration of adenosine through a central line, or administration of adenosine prior to emergency department arrival or in a patient care area other than the emergency department (including pre-hospital administration).

Definitions

Successful first-dose conversion was defined as the conversion of PSVT to sinus rhythm (as confirmed on ECG) after a single dose of adenosine was administered. Adverse events were defined as either subsequent arrhythmia or chest pain.

Data collection/data analysis

Given that adenosine was administered at standard doses of 6mg for the majority of patients (some received higher initial doses due to alternative circumstances), and patient weights were varied, a natural experiment existed that generated two cohorts: those who received an initial dose of adenosine ≥0.1mg/kg or <0.1mg/kg. Baseline data collected about all patients included dose administered, weight, gender, age, heart rate prior to adenosine, documentation of vagal maneuvers, comorbidities (congestive heart failure, history of PSVT, cardiac structural abnormality), prior to admission medications (caffeine, stimulant, calcium channel blocker, beta blocker, or anti-arrhythmic), and prior to adenosine labs (magnesium, calcium, potassium, troponin).

Data analysis was performed using Stata 16.1 (StataCorp, College Station, TX). The primary outcome for analysis was the successful conversion of PSVT to sinus rhythm with the first dose of adenosine. Significance testing was performed using chi-squared for categorical values with the exception of Fisher’s exact test for the outcome of adverse events because the frequency in cells was less than five. T-test was used for continuous variables. The threshold for significance was set at 0.05. No a priori power calculation was done as the analysis included all available charts. Post-hoc power calculations showed that the study had an 80% power to detect a difference of 20.5% in conversion rate, given a baseline conversion rate of 78% from prior studies and n=24 in the experimental higher dose group.

Data source/ethics

Patients were identified for potential inclusion based on data generated from Stanford Research Repository (STARR) reporting systems, which provided a list of patients who both had adenosine ordered and were located within the emergency department. This study was approved by the Stanford University Institutional Research Board (IRB) on September 27, 2019, under eProtocol #53026.

This research used data or services provided by STARR, “Stanford medicine Research data Repository,” a clinical data warehouse containing live Epic data from Stanford Health Care, the Stanford Children’s Hospital, the University Healthcare Alliance, Packard Children's Health Alliance clinics and other auxiliary data from Hospital applications such as radiology PACS. STARR platform is developed and operated by the Stanford Medicine Research IT team and is made possible by the Stanford School of Medicine Research Office.

Data was collected and maintained securely in the REDCap platform. The Stanford REDCap platform (http://redcap.stanford.edu) is developed and operated by the Stanford Medicine Research IT team. The REDCap platform services at Stanford are subsidized by a) the Stanford School of Medicine Research Office, and b) the National Center for Research Resources and the National Center for Advancing Translational Sciences, National Institutes of Health, through grant UL1 TR001085.

## Results

During the study period, 364 records were reviewed and 76 patients qualified for inclusion in the study, all of whom were treated according to the standard of care in the emergency department during the study period - the 2015 AHA/ACC/HRS Guidelines for the Management of Adult Patients with Supraventricular Tachycardia.

Due to varied patient weights, twenty-four patients received a dose of adenosine ≥0.1mg/kg, and 52 patients received a dose of <0.1mg/kg. Their characteristics are displayed in Table 1. The mean weight-based dose in the higher dose group was 0.12mg/kg, while the mean weight-based dose in the lower dose group was 0.07mg/kg (p<0.001), with a mean weight of 91 kg in the higher dose group, and a mean weight of 61.7 kg in the lower dose group (p<0.001). Characteristics were similar between both groups with the exception that patients in the higher dose cohort were significantly more likely than patients in the lower dose cohort to be female (91.7% vs 46.2%, p<0.001), and to have a prior history of PSVT (75.0% vs. 48.1%, p=0.028).

**Table 1:** Patient Characteristics mg: milligram kg: kilogram SD: standard deviation n: number Bpm: beats per minute CHF: congestive heart failure PSVT: paroxysmal supraventricular tachycardia ng: nanogram dL: deciliter mL: milliliter mmol: millimole *: Indicates t-test used for significance; all others used chi-squared

Variable	Higher (>0.1mg/kg) Dose (n=24)	Lower (<0.1mg/kg) Dose (n=52)	P-value
Dose (mg/kg), mean (SD)	0.12 (0.02)	0.07 (0.02)	<0.001*
Weight (kg), mean (SD)	91.0 (29.1)	61.7 (15.0)	<0.001*
Female Gender, n (%)	22 (91.7)	24 (46.2)	<0.001
Age, mean (SD)	58.5 (17.7)	64.3 (16.5)	0.19*
Heart Rate Prior to Adenosine (bpm), mean (SD)	173.3 (24.3)	171.7 (25.2)	0.80*
Vagal Maneuvers Documented, n (%)	9 (37.5)	22 (42.3)	0.69
Comorbidities			
CHF, n (%)	2 (8.3)	9 (17.3)	0.30
History of PSVT, n (%)	18 (75.0)	25 (48.1)	0.028
Cardiac Structural Abnormality	3 (12.5)	8 (15.3)	0.74
Prior to Admission Medications			
Caffeine, n (%)	1 (4.2)	2 (3.9)	0.95
Stimulant, n (%)	1 (4.2)	4 (7.7)	0.56
Calcium Channel Blocker, n (%)	5 (20.8)	6 (11.5)	0.28
Beta Blocker, n (%)	7 (29.2)	14 (26.9)	0.84
Anti-Arrhythmic, n (%)	1 (4.2)	5 (9.6)	0.41
Labs Prior to Adenosine			
Magnesium (mg/dL), mean (SD)	2.16 (0.2)	2.1 (0.2)	0.44*
Calcium (mg/dL), mean (SD)	9.41 (0.4)	9.3 (0.6)	0.46*
Potassium (mmol/dL), mean (SD)	4.15 (0.5)	4.2 (0.9)	0.6*
Elevated Troponin I (≥0.04 ng/mL), n (%)	5 (20.8)	12 (23.1)	0.83

The outcomes associated with adenosine dosing in both cohorts are summarized in Table 2. The higher-dose adenosine cohort had a significantly higher first-dose success rate of 92.7% as compared to the 59.6% success rate seen in the lower-dose cohort (p=0.006). There was no significant difference seen in adverse event rates between the two groups (8.3% in the higher dose group vs. 3.9% in the lower dose group, p=0.75). Two adverse events were seen in each group: one subsequent arrhythmia and one episode of chest pain in the higher dose cohort, and two subsequent arrhythmias in the lower dose cohort.

**Table 2:** Outcomes of First Dose of Adenosine mg: milligrams kg: kilograms n: number †: p-value calculated with chi-squared *: p-value calculated with Fisher’s exact

	Higher (≥0.1mg/kg) Dose (n=24)	Lower (<0.1mg/kg) Dose (n=52)	P-value
Successful Conversion, n (%)	22 (92.7)	31 (59.6)	0.006^†^
Adverse Effects, n (%)	2 (8.3)	2 (3.9)	0.75*

## Discussion

Overall, this represents the institutional experience of a single major academic medical center over nearly 5 years. When the higher dose (≥0.1mg/kg) and lower dose (<0.1mg/kg) cohorts were compared, those in the higher dose cohort were more likely to be female and to weigh less. These variables are connected and the asymmetrical distribution between cohorts was most likely the byproduct of the natural experimental design. Since the majority of patients received a fixed dose of 6mg, the smaller average mass of females would make them more likely to appear in the higher-dose cohort.

Documented history of PSVT was also significantly higher in the higher-dose cohort as compared to the lower-dose cohort. This may also tie into the gender discrepancy explained above as women are more likely to be affected by PSVT and tend to present at an earlier age [13].

The primary finding of the study was that the overall rate of first-dose pharmacological conversion was statistically greater in the higher-dose cohort when compared to the lower-dose cohort (92.7% vs. 59.6%, p=0.006). As all patients included in the analysis had PSVT confirmed by a cardiologist over-read following the ED encounter, this confirms that the effect was not due to other types of arrhythmias. The rates of adverse effects were not higher in the higher dose group, suggesting that this increased efficacy did not come at an increased risk to patients. Care should be taken in interpreting these results as adverse events were rare and the overall study size was relatively small.

Current guidelines for the treatment of adult PSVT with adenosine are based on a study that did use varying doses of adenosine (3mg, 6mg, 9mg, 12mg) but not weight-based dosing [15, 16]. The existing evidence base from the pediatric literature suggests a 0.1mg/kg weight-based starting dose [2,15]. The only other study looking at weight-based conversion rates reported that patients were more likely to convert to sinus rhythm with higher weight-based doses of adenosine, but did not evaluate 0.1mg/kg dosing [14]. Our study’s finding of superior efficacy when using higher weight-based initial dosing of adenosine for PSVT in adults without any increase in adverse event rate, in conjunction with the limited existing evidence base helps generate the hypothesis that a 0.1mg/kg weight-based starting dose may be more desirable than a fixed 6mg starting dose for adults. Future randomized, controlled trials could further explore the effect of weight-based dosing of adenosine for PSVT and more definitively determine if the recommended initial adenosine dose for PSVT in adults should be revised.

This study has several limitations. It is a single-center study that limits external validity and is a retrospective chart review, which may underestimate adverse effects. Additionally, when cohorts were stratified by weight-based dose, more patients were found to be female, lighter in weight, and have a history of PSVT, resulting in unbalanced baseline characteristics. While this unbalance was expected due to how the cohorts were derived, it may mask an important role that age or gender plays in adenosine dosing that has not been previously seen.

## Conclusions

These findings support the hypothesis that adenosine, when given at doses greater than or equal to 0.1mg/kg, may be more effective than doses less than 0.1mg/kg for PSVT. These hypothesis-generating findings provide the basis for a subsequent randomized controlled trial to investigate weight-based versus standard dosing of adenosine for the treatment of PSVT.
